# Supplementary material for: HIVprotI: an integrated web based platform for prediction and design of HIV proteins inhibitors
Source: J Cheminform. 2018 Mar 9;10:12. doi: 10.1186/s13321-018-0266-y (PMC5845081; doi:10.1186/s13321-018-0266-y)
Supplement: Supplementary file 2 — Additional file 2. Source code of HIVProtI web server. [file 13321_2018_266_MOESM2_ESM.zip › HIVprotI_Source-code/hivprotI/weka-3-6-6/doc/weka/core/converters/TextDirectoryLoader.html]

TextDirectoryLoader


---


|  |  |  |
| --- | --- | --- |
| **Overview** \| **Package** \| **Class** \| **Tree** \| **Deprecated** \| **Index** \| **Help** \| **Weka's home** | |  |
| **PREV CLASS**   **NEXT CLASS** | **FRAMES**    **NO FRAMES**     **All Classes** |  |
| SUMMARY: NESTED \| FIELD \| CONSTR \| METHOD | DETAIL: FIELD \| CONSTR \| METHOD |  |


---


## weka.core.converters Class TextDirectoryLoader

```
java.lang.Object
  weka.core.converters.AbstractLoader
      weka.core.converters.TextDirectoryLoader
```

**All Implemented Interfaces:**: java.io.Serializable, BatchConverter, Loader, OptionHandler, RevisionHandler

---

``` public class TextDirectoryLoader extends AbstractLoader implements BatchConverter, OptionHandler ```

Loads all text files in a directory and uses the subdirectory names as class labels. The content of the text files will be stored in a String attribute, the filename can be stored as well.


Valid options are:

```
 -D
  Enables debug output.
  (default: off)
```

```
 -F
  Stores the filename in an additional attribute.
  (default: off)
```

```
 -dir <directory>
  The directory to work on.
  (default: current directory)
```

Based on code from the TextDirectoryToArff tool:

- Original tool
- Current version
- Wiki article

**Version:**
:   $Revision: 7088 $

**Author:**
:   Ashraf M. Kibriya (amk14 at cs.waikato.ac.nz), Richard Kirkby (rkirkby at cs.waikato.ac.nz), fracpete (fracpete at waikato dot ac dot nz)

**See Also:**: `Loader`, Serialized Form

---

**Field Summary**

**Fields inherited from interface weka.core.converters.Loader**

`BATCH, INCREMENTAL, NONE`


**Constructor Summary**

`TextDirectoryLoader()`
  
          default constructor


| **Method Summary** | |
| --- | --- |
| `java.lang.String` | `charSetTipText()`             the tip text for this property |
| `java.lang.String` | `debugTipText()`             the tip text for this property |
| `java.lang.String` | `getCharSet()`             Get the character set to use when reading text files. |
| `Instances` | `getDataSet()`             Return the full data set. |
| `boolean` | `getDebug()`             Gets whether additional debug information is printed. |
| `java.io.File` | `getDirectory()`             get the Dir specified as the source |
| `java.lang.String` | `getFileDescription()`             Returns a description of the file type, actually it's directories. |
| `Instance` | `getNextInstance(Instances structure)`             TextDirectoryLoader is unable to process a data set incrementally. |
| `java.lang.String[]` | `getOptions()`             Gets the setting |
| `boolean` | `getOutputFilename()`             Gets whether the filename will be stored as an extra attribute. |
| `java.lang.String` | `getRevision()`             Returns the revision string. |
| `Instances` | `getStructure()`             Determines and returns (if possible) the structure (internally the header) of the data set as an empty set of instances. |
| `java.lang.String` | `globalInfo()`             Returns a string describing this loader |
| `java.util.Enumeration` | `listOptions()`             Lists the available options |
| `static void` | `main(java.lang.String[] args)`             Main method. |
| `java.lang.String` | `outputFilenameTipText()`             the tip text for this property |
| `void` | `reset()`             Resets the loader ready to read a new data set |
| `void` | `setCharSet(java.lang.String charSet)`             Set the character set to use when reading text files (an empty string indicates that the default character set will be used). |
| `void` | `setDebug(boolean value)`             Sets whether to print some debug information. |
| `void` | `setDirectory(java.io.File dir)`             sets the source directory |
| `void` | `setOptions(java.lang.String[] options)`             Parses a given list of options. |
| `void` | `setOutputFilename(boolean value)`             Sets whether the filename will be stored as an extra attribute. |
| `void` | `setSource(java.io.File dir)`             Resets the Loader object and sets the source of the data set to be the supplied File object. |

**Methods inherited from class weka.core.converters.AbstractLoader**

`setRetrieval, setSource`

**Methods inherited from class java.lang.Object**

`equals, getClass, hashCode, notify, notifyAll, toString, wait, wait, wait`

**Constructor Detail**

### TextDirectoryLoader

```
public TextDirectoryLoader()
```

:   default constructor


**Method Detail**

### globalInfo

```
public java.lang.String globalInfo()
```

:   Returns a string describing this loader

    :   **Returns:**: a description of the evaluator suitable for displaying in the explorer/experimenter gui

---


### listOptions

```
public java.util.Enumeration listOptions()
```

:   Lists the available options

    :   **Specified by:**: `listOptions` in interface `OptionHandler`
    :   **Returns:**: an enumeration of the available options

---


### setOptions

```
public void setOptions(java.lang.String[] options)
                throws java.lang.Exception
```

:   Parses a given list of options.


    Valid options are:

    ```
     -D
      Enables debug output.
      (default: off)
    ```

    ```
     -F
      Stores the filename in an additional attribute.
      (default: off)
    ```

    ```
     -dir <directory>
      The directory to work on.
      (default: current directory)
    ```

    :   **Specified by:**: `setOptions` in interface `OptionHandler`
    :   **Parameters:**: `options` - the options **Throws:**: `java.lang.Exception` - if options cannot be set

---


### getOptions

```
public java.lang.String[] getOptions()
```

:   Gets the setting

    :   **Specified by:**: `getOptions` in interface `OptionHandler`
    :   **Returns:**: the current setting

---


### charSetTipText

```
public java.lang.String charSetTipText()
```

:   the tip text for this property

    :   **Returns:**: the tip text

---


### setCharSet

```
public void setCharSet(java.lang.String charSet)
```

:   Set the character set to use when reading text files (an empty string
    indicates that the default character set will be used).

    :   **Parameters:**: `charSet` - the character set to use.

---


### getCharSet

```
public java.lang.String getCharSet()
```

:   Get the character set to use when reading text files. An empty
    string indicates that the default character set will be used.

    :   **Returns:**: the character set name to use (or empty string to indicate that the default character set will be used).

---


### setDebug

```
public void setDebug(boolean value)
```

:   Sets whether to print some debug information.

    :   **Parameters:**: `value` - if true additional debug information will be printed.

---


### getDebug

```
public boolean getDebug()
```

:   Gets whether additional debug information is printed.

    :   **Returns:**: true if additional debug information is printed

---


### debugTipText

```
public java.lang.String debugTipText()
```

:   the tip text for this property

    :   **Returns:**: the tip text

---


### setOutputFilename

```
public void setOutputFilename(boolean value)
```

:   Sets whether the filename will be stored as an extra attribute.

    :   **Parameters:**: `value` - if true the filename will be stored in an extra attribute

---


### getOutputFilename

```
public boolean getOutputFilename()
```

:   Gets whether the filename will be stored as an extra attribute.

    :   **Returns:**: true if the filename is stored in an extra attribute

---


### outputFilenameTipText

```
public java.lang.String outputFilenameTipText()
```

:   the tip text for this property

    :   **Returns:**: the tip text

---


### getFileDescription

```
public java.lang.String getFileDescription()
```

:   Returns a description of the file type, actually it's directories.

    :   **Returns:**: a short file description

---


### getDirectory

```
public java.io.File getDirectory()
```

:   get the Dir specified as the source

    :   **Returns:**: the source directory

---


### setDirectory

```
public void setDirectory(java.io.File dir)
                  throws java.io.IOException
```

:   sets the source directory

    :   **Parameters:**: `dir` - the source directory **Throws:**: `java.io.IOException` - if an error occurs

---


### reset

```
public void reset()
```

:   Resets the loader ready to read a new data set

    :   **Specified by:**: `reset` in interface `Loader` **Overrides:**: `reset` in class `AbstractLoader`

---


### setSource

```
public void setSource(java.io.File dir)
               throws java.io.IOException
```

:   Resets the Loader object and sets the source of the data set to be
    the supplied File object.

    :   **Specified by:**: `setSource` in interface `Loader` **Overrides:**: `setSource` in class `AbstractLoader`
    :   **Parameters:**: `dir` - the source directory. **Throws:**: `java.io.IOException` - if an error occurs

---


### getStructure

```
public Instances getStructure()
                       throws java.io.IOException
```

:   Determines and returns (if possible) the structure (internally the
    header) of the data set as an empty set of instances.

    :   **Specified by:**: `getStructure` in interface `Loader` **Specified by:**: `getStructure` in class `AbstractLoader`
    :   **Returns:**: the structure of the data set as an empty set of Instances **Throws:**: `java.io.IOException` - if an error occurs

---


### getDataSet

```
public Instances getDataSet()
                     throws java.io.IOException
```

:   Return the full data set. If the structure hasn't yet been determined
    by a call to getStructure then method should do so before processing
    the rest of the data set.

    :   **Specified by:**: `getDataSet` in interface `Loader` **Specified by:**: `getDataSet` in class `AbstractLoader`
    :   **Returns:**: the structure of the data set as an empty set of Instances **Throws:**: `java.io.IOException` - if there is no source or parsing fails

---


### getNextInstance

```
public Instance getNextInstance(Instances structure)
                         throws java.io.IOException
```

:   TextDirectoryLoader is unable to process a data set incrementally.

    :   **Specified by:**: `getNextInstance` in interface `Loader` **Specified by:**: `getNextInstance` in class `AbstractLoader`
    :   **Parameters:**: `structure` - ignored **Returns:**: never returns without throwing an exception **Throws:**: `java.io.IOException` - always. TextDirectoryLoader is unable to process a data set incrementally.

---


### getRevision

```
public java.lang.String getRevision()
```

:   Returns the revision string.

    :   **Specified by:**: `getRevision` in interface `RevisionHandler`
    :   **Returns:**: the revision

---


### main

```
public static void main(java.lang.String[] args)
```

:   Main method.

    :   **Parameters:**: `args` - should contain the name of an input file.


---


|  |  |  |
| --- | --- | --- |
| **Overview** \| **Package** \| **Class** \| **Tree** \| **Deprecated** \| **Index** \| **Help** \| **Weka's home** | |  |
| **PREV CLASS**   **NEXT CLASS** | **FRAMES**    **NO FRAMES**     **All Classes** |  |
| SUMMARY: NESTED \| FIELD \| CONSTR \| METHOD | DETAIL: FIELD \| CONSTR \| METHOD |  |


---
